# FUNCTIONAL LIMITATION IN LATER LIFE: THE IMPACT OF SIPS, SOCIALIZATION, AND SADNESS

**DOI:** 10.1093/geroni/igz038.2089

**Published:** 2019-11-08

**Authors:** Rosanna Scott, Chelsea Wiener, Daniel Paulson

**Affiliations:** University of Central Florida, Orlando, Florida, United States

## Abstract

Recent studies posit discrepant impacts of alcohol use on health outcomes. Potential reasons for contrasting results include: (1) selection bias involved in classifying individuals as “abstainers” or “drinkers,” (2) unexamined demographic variables associated with alcohol use, and (3) unaddressed mechanisms of action. Given new studies identifying socialization as a mediator between alcohol use and health outcomes, this study examines social interaction and depressive symptoms, respectively, as serial mediators in the relationship between moderate alcohol use and functional limitation, while employing methods to reduce selection bias. HRS data from 2012 and 2014 were utilized (n=1,902); heavy drinkers, adults younger than 65, and respondents with inconsistent alcohol use from 2008 to 2014 were excluded. Hypotheses were evaluated using a longitudinal serial mediation model with bias-corrected bootstrapping. Results indicated that, in the context of demographic variables, medical burden, and previous functional limitation, the beneficial relationship between moderate alcohol use and future functional limitation is only present when considering social interaction and depressive symptoms as mediators, both individually and serially (variance accounted for=39.4%). There was no direct effect of moderate alcohol use on functional limitation outside the context of these mediators. Data indicate that previously suggested relationships between moderate drinking and reduced functional limitation are better explained through increased social interaction and subsequent reduced depressive symptoms. Results identify social interaction as an accessible treatment target to prevent/reduce depressive symptoms and functional limitation in later-life, and support increased assessment of IADLs in adults experiencing depressive symptoms to facilitate early treatment/prevention of functional limitation.

